# Lower, more frequent cisplatin dosing minimizes hearing loss in head and neck cancer

**DOI:** 10.1172/jci.insight.196230

**Published:** 2025-10-22

**Authors:** Katharine A. Fernandez, Abu S. Chowdhury, Amanda Bonczkowski, Paul D. Allen, Maura H. Campbell, David S. Lee, Charvi Malhotra, Brandi R. Page, Deborah A. Mulford, Candice Evita Ortiz, Peter L. Santa Maria, Peter Kullar, Saad A. Khan, Shawn D. Newlands, Nicole C. Schmitt, Lisa L. Cunningham

**Affiliations:** 1 National Institute on Deafness and Other Communication Disorders, Bethesda, Maryland, USA.; 2 Department of Otolaryngology, University of Rochester Medical Center, Rochester, New York, USA.; 3 Department of Otolaryngology — Head and Neck Surgery, Washington University in St. Louis, St. Louis, Missouri, USA.; 4 Stanford University, Division of Oncology, Stanford, California, USA.; 5 Department of Radiation Oncology and Molecular Sciences, Johns Hopkins University, Baltimore, Maryland, USA.; 6 Wilmot Cancer Institute, University of Rochester Medical Center, Rochester, New York, USA.; 7 Capital Institute of Hearing and Balance, Silver Spring, Maryland, USA.; 8 Department of Otolaryngology, University of Pittsburgh, Pittsburgh, Pennsylvania, USA.; 9 Manchester Foundation Trust, Manchester, United Kingdom.; 10 Department of Otolaryngology — Head and Neck Surgery, Emory University, Atlanta, Georgia, USA.

**Keywords:** Clinical Research, Oncology, Clinical practice, Head and neck cancer, Outcomes research

## Abstract

**BACKGROUND:**

Cisplatin is often the cytotoxic drug of choice for chemoradiation therapy (CRT) for head and neck squamous cell carcinoma (HNSCC), but it can lead to irreversible hearing loss. There may be similar oncologic outcomes but different toxicity profiles depending on whether cisplatin is given at 75–100 mg/m^2^ every 3 weeks or 30–40 mg/mg^2^ weekly. This study compares cisplatin-induced hearing loss in patients with HNSCC receiving similar cumulative doses of cisplatin administered either on higher-dose or lower-dose treatment schedules.

**METHODS:**

Using the Enhancing Cancer Hearing Outcomes (ECHO) dataset from 5 academic centers, we conducted a multicenter retrospective cohort study of adults (≥18 years) with HNSCC receiving cisplatin-based CRT. Participants were grouped by cisplatin dose schedule: every 3 weeks (≥75 mg/m²) or weekly (<75 mg/m²). Hearing loss was assessed using American Speech-Language-Hearing Association (ASHA) and Common Terminology Criteria for Adverse Events (CTCAE) v5.0 threshold shift criteria based on audiograms obtained ≤120 days before and after treatment. Risk differences and predictors of hearing loss were evaluated using χ^2^ analyses and multivariate regression. Kaplan-Meier curves assessed overall and disease-free survival.

**RESULTS:**

Among 564 participants (1,127 ears), lower-dose weekly cisplatin was associated with significantly lower incidence of hearing loss (ASHA criteria: 57% vs. 82%; CTCAE criteria: 39% vs. 69%). CTCAE grade ≥2 hearing loss occurred in 18% of the weekly group versus 50% of the 3-week group. Multivariate analysis confirmed treatment schedule as an independent predictor of ototoxicity. Two-year survival outcomes did not differ between groups.

**CONCLUSIONS:**

Weekly low-dose cisplatin significantly reduced the incidence and severity of hearing loss without compromising survival, supporting its broader use in CRT for HNSCC.

## Abstract 2

Weekly low-dose cisplatin causes less hearing loss than higher triweekly doses, with no difference in survival, suggesting a safer treatment option for head and neck cancer.

## Introduction

Cisplatin has been an essential medication for treatment of various cancers since its approval by the FDA in the 1970s. Cisplatin functions in part by forming platinum-DNA adducts that disrupt DNA replication and transcription, ultimately leading to cancer cell apoptosis (1, 2). Despite its potent antitumor effects, cisplatin’s therapeutic utility is often compromised by its significant side effect profile, including ototoxicity, which manifests as irreversible hearing loss (3–5).

Ototoxicity refers to drug-induced damage to the inner ear, affecting both the cochlea and vestibular system, resulting in hearing loss, tinnitus, and balance disorders. Cisplatin-induced ototoxicity is dose dependent, often more severe with higher cumulative doses, and can significantly impact quality of life for cancer survivors (6, 7). Understanding the balance between achieving optimal oncologic outcomes and minimizing adverse effects is crucial for improving patient outcomes.

The therapeutic landscape for HNSCC has seen a growing interest in optimizing cisplatin dosing strategies to mitigate adverse effects while maintaining efficacy. Studies have explored various cisplatin dosing regimens, comparing the standard higher dose (100 mg/m² every 3 weeks [“triweekly”]) with weekly lower-dose administrations (30–40 mg/m²) to determine whether reduced intensity regimens can mitigate toxicity without compromising oncologic outcomes. Several studies have demonstrated that weekly cisplatin dosing, compared with the standard triweekly regimen, offers similar efficacy in terms of tumor control, disease-free survival (DSF), and overall survival (OS), while exhibiting a more favorable toxicity profile. Across both definitive and postoperative treatment settings, weekly administration has been associated with reduced rates of mucositis, nephrotoxicity, hematologic toxicity, and treatment interruptions, without compromising oncologic outcomes (8–14). These findings were further supported by a recent randomized trial demonstrating the noninferiority of weekly cisplatin for OS and improved tolerability (15).

Despite these data indicating that lower weekly-dose cisplatin regimens are generally less toxic and noninferior to higher triweekly-dose regimens, the relationship between cisplatin dose and ototoxicity remains unclear. High cumulative doses are well-documented risk factors for severe hearing loss (16); however, even low doses can lead to significant ototoxicity in susceptible individuals. This highlights the need for a deeper understanding of how different dosing regimens influence hearing outcomes, especially in patients with preexisting risk factors for hearing loss.

Here, we aimed to provide a more comprehensive understanding of the impact of low- versus high-dose cisplatin dosing regimens on hearing loss. Our goal was to inform clinical decisions that optimize therapeutic efficacy while preserving quality of life for patients with head and neck squamous cell cancer (HNSCC).

## Results

### Characteristics of the clinical data set.

**Figure 1:**
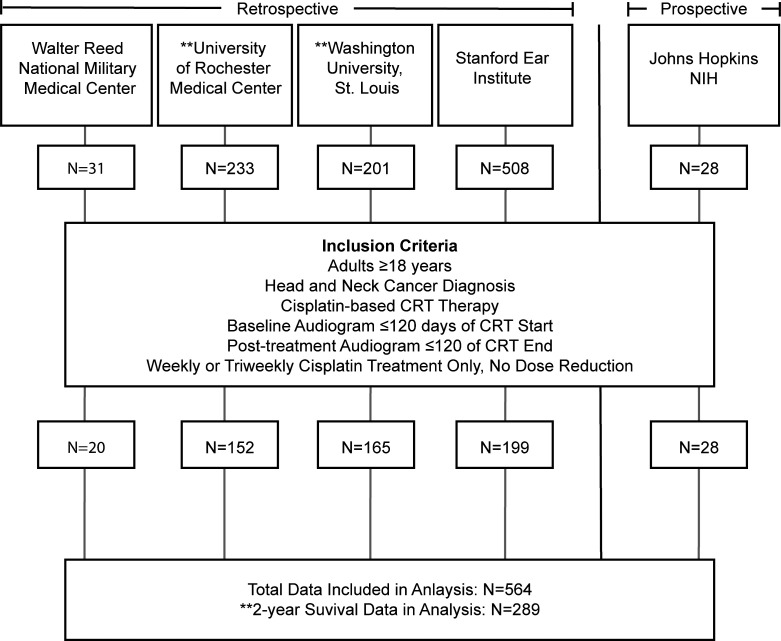
Schematic diagram of the ECHO dataset. Data were collected from 5 US-based academic and clinical centers. The dataset was filtered to include only adults (≥18 years) with confirmed HNSCC who received CRT on a consistent weekly or triweekly schedule. Patients whose dosing schedules were modified (e.g., from triweekly to weekly) were excluded. After filtering, 564 individuals remained, of whom 289 had survival data available through 2 years of follow-up.

Retrospective clinical data were compiled for a total of 564 participants, all of whom met study inclusion criteria (Figure 1): patients ≥18 years of age, diagnosed with head and neck squamous cell carcinoma (HNSCC) and treated with cisplatin-based chemoradiation therapy (CRT). Participants were excluded if they had non-HNSCC cancers, insufficient clinical or treatment data, lacked a valid audiogram within 120 days before and after treatment, or presented with a bilateral profound hearing loss at baseline (>90 dB HL average threshold at 1, 2, and 4 kHz) (17).

Participants were categorized by cisplatin treatment regimen: 228 (40%) received low-dose cisplatin (<75 mg/m^2^) and 336 (60%) received triweekly high-dose cisplatin (≥75 mg/m^2^) (Table 1). To maintain consistency in treatment exposure, patients who underwent intratherapy dose reductions or schedule changes were excluded from the analysis. The cohort’s mean age was 59 years (IQR, 52–64), with the low-dose group slightly older than the high-dose group (median age 60 vs. 58 years; Wilcoxon’s 2-sample test, *P* < 0.02). Across both groups, there was a predominance of males (≥79%) and near-universal use of concurrent radiation therapy (98%). Most tumors were localized to the oropharynx (52%), followed by the oral cavity (16%), larynx (7%), nasopharynx (13%), hypopharynx (2%), and other sites (10%), including salivary glands, maxilla, mandible, sinuses, and pharynx. Fewer than 1% had tumors of unknown primary origin. A Wilcoxon’s 2-sample test indicated a significant difference in tumor site distribution between the treatment groups, driven largely by the higher prevalence of oropharyngeal tumors in the high-dose group (*P* < 0.01).

The overall median cumulative cisplatin dose was 200 mg/m^2^. However, cumulative dose distributions differed significantly between treatment groups (χ^2^, *P* < 0.01), with low-dose, weekly regimens ranging from 30 to 520 mg/m^2^ and high-dose, triweekly regimens ranging from 75 to 540 mg/m^2^.

Baseline hearing status was assessed per ear using pure-tone average (PTA) at 0.5, 1, 2, and 4 kHz. Across the full cohort and within subgroups, most ears (≥85%) exhibited normal hearing or mild hearing loss at baseline. The low-dose group showed a significantly higher incidence of moderate hearing loss, resulting in a statistically significant difference in baseline hearing status distribution between treatment regimens (Wilcoxon’s 2-sample test, *P* < 0.0001)

### Individuals receiving lower-dose, weekly cisplatin experience less cisplatin-induced hearing loss than those treated with higher-dose, triweekly cisplatin.

In total, 1,127 ears of 564 participants were included in the analyses. The primary outcome was the incidence of Common Terminology Criteria for Adverse Events–defined (CTCAE-defined) hearing change, based on differences in auditory thresholds (“threshold shifts”) between baseline and posttreatment audiograms.

**Figure 2:**
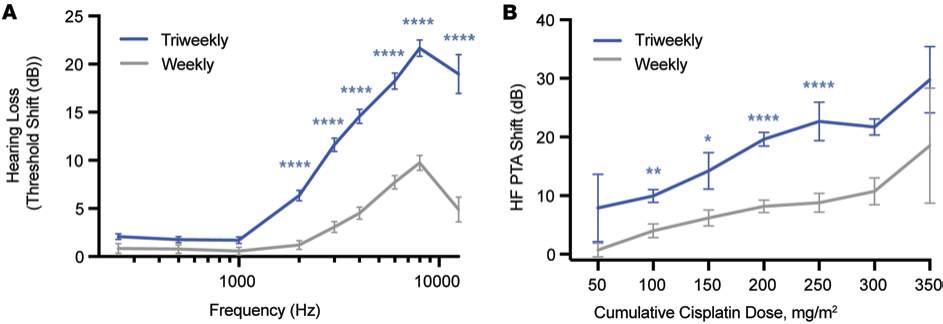
Weekly cisplatin administration is associated with reduced auditory threshold shifts. Audiometric thresholds collected before and after cisplatin treatment were used to determine cisplatin-induced hearing loss (threshold shifts). (**A**) High-dose, triweekly cisplatin administration (*n* = 654 ears) resulted in significant hearing loss from 2 to 12.5 kHz, ranging on average from 6 to 21 dB. In contrast, low-dose, weekly cisplatin administration (*n* = 454 ears) resulted in significantly less cisplatin-induced hearing loss at 2–12.5 kHz, ranging on average 1–10 dB. Data are presented as mean ± SEM; 2-way ANOVA, Šidák’s multiple-comparison post hoc analysis. (**B**) The impact of cumulative cisplatin dose on high-frequency (4–12.5 kHz) hearing loss was assessed per treatment schedule. When cumulative cisplatin dose was comparable across groups (dose bin = 50 mg/m^2^), individuals receiving high-dose, triweekly cisplatin consistently experienced significantly greater hearing loss (threshold shifts). Data are presented as mean ± SEM; 2-way ANOVA; Šidák’s multiple-comparison post hoc analysis. *P < 0.05, **P < 0.01, ****P < 0.0001.

We began by evaluating threshold shift data as a function of cisplatin treatment schedule. Two-way ANOVA revealed a significant effect of dose schedule (weekly low-dose vs. triweekly high-dose) on threshold shifts (*F_1,8727_* = 401.3, *P* < 0.0001), as well as a significant effect of frequency (*F_8,8727_* = 145.9, *P* < 0.0001) (Figure 2A). There was also a significant interaction between these factors (*F_8,8727_* = 25.97, *P* < 0.0001). Šidák’s multiple-comparison post hoc analysis demonstrated that individuals receiving low-dose, weekly cisplatin had significantly smaller threshold shifts at frequencies ≥2 kHz (*P* < 0.0001). The largest differences were observed at 8 kHz, with average shifts of 21.7 ± 21.5 dB in the triweekly group compared with 9.7 ± 15.7 dB in the weekly group. These data indicate that participants receiving high-dose, triweekly cisplatin treatment experienced significantly more cisplatin-induced hearing loss compared with those on the low-dose weekly treatment schedule.

Cumulative cisplatin doses ranged from 30 to 540 mg/m² across the cohort. We next assessed the relationship between cumulative dose and high-frequency hearing loss (PTA at 4, 6, 8, and 12.5 kHz) within each treatment group (Figure 2B). High-frequency hearing loss increased significantly with cumulative dose (*F*_6,1064_ = 7.396, *P* < 0.001), but the effect varied by treatment schedule. At comparable cumulative doses, weekly low-dose cisplatin was associated with significantly less hearing loss than triweekly high-dose cisplatin (*F_1,1064_* = 22.27, *P* < 0.0001), particularly at cumulative doses of 100 (*P* < 0.05; weekly *n* = 78, triweekly *n* = 134), 150 (*P* < 0.05; weekly *n* = 75, triweekly *n* = 35), 200 (*P* < 0.0001; weekly *n* = 155, triweekly *n* = 212), and 250 mg/m² (*P* < 0.001; weekly *n* = 98, triweekly *n* = 28) (Šidák’s post hoc analysis). These data indicate that when the cumulative dose of cisplatin was equivalent, participants receiving triweekly cisplatin had significantly more high-frequency hearing loss compared with those receiving the weekly regimen.

To further investigate predictors of cisplatin-induced hearing loss, we performed an ordinary least squares (OLS) regression using standard PTA threshold shift data at 0.5, 1, 2, and 4 kHz. Controlling for age, sex, cumulative cisplatin dose, and baseline hearing, cisplatin dose schedule remained a significant predictor of hearing loss severity (PROC REG, SAS; β = 4.51, 95% CI (3.33, 6.69), *P* < 0.0001; Table 2). Additional significant predictors included age (β = 0.16, 95% CI (0.11, 0.22), *P* < 0.0001), cumulative cisplatin dose (β = 0.03, 95% CI (0.03, 0.04), *P* < 0.0001), and baseline hearing (β = –0.17, 95% CI (–0.21, –0.12), *P* < 0.0001).

Together, these results demonstrate that individuals treated with low-dose, weekly cisplatin had significantly less cisplatin-induced hearing loss than those receiving triweekly cisplatin.

### Weekly cisplatin administration is associated with reduced incidence and severity of cisplatin-induced hearing loss.

**Figure 3:**
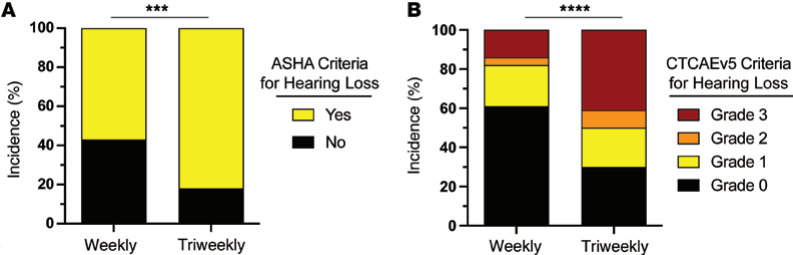
Weekly cisplatin is associated with reduced incidence and severity of cisplatin-induced hearing loss, per ASHA and CTCAE criteria. ASHA and CTCAE criteria for clinically meaningful changes in hearing loss were applied to threshold shift data. (**A**) The incidence of cisplatin-induced hearing loss among individuals receiving weekly cisplatin (*n* = 228), per ASHA criteria, was 57%. Individuals receiving triweekly cisplatin (*n* = 336) had a significantly higher incidence (82% triweekly) of cisplatin-induced hearing loss. Data are presented as percentage of individuals per group. χ^2^ analysis. (**B**) Low-dose, weekly cisplatin administration was associated with reduced incidence and severity of cisplatin-induced hearing loss, per CTCAE criteria. χ^2^ analysis. Analysis showed a significant difference in the distribution of CTCAE hearing loss grades, wherein the incidence of a grade ≥2 was reduced from 50% in the high-dose, triweekly group to 18% in the low-dose, weekly group. Data represent the percentage of individuals per group. **P < 0.01, ***P < 0.001, ****P < 0.0001.

We applied American Speech-Language-Hearing Association (ASHA; 1994) and CTCAE criteria to evaluate both the incidence and severity of hearing loss. These criteria are designed to assess changes in hearing likely to be clinically meaningful. Among individuals receiving triweekly cisplatin, the incidence of clinically meaningful hearing loss was 82% per ASHA and 69% per CTCAE grade ≥1 criteria (Figure 3, A and B). In contrast, individuals treated with weekly cisplatin showed significantly lower incidence of hearing loss: 57% per ASHA criteria (χ^2^(1) = 14.74, *P* < 0.0001) and 39% per CTCAE criteria (χ^2^(1) = 19.38, *P* < 0.0001). These findings indicate that the incidence of cisplatin-induced hearing loss was significantly reduced with low-dose, weekly cisplatin relative to the triweekly regimen.

Beyond incidence, the CTCAE system grades hearing loss severity on a scale from 1 (mild) to 4 (profound), where grade 1 reflects a mild change not typically requiring intervention and grade 2 or higher reflects moderate to severe hearing loss warranting intervention. The incidence of hearing loss with CTCAE grade ≥2 was significantly lower in the low-dose, weekly group (18%) compared with the triweekly group (50%) (χ^2^(1) = 25.61, *P* < 0.0001) (Figure 3B). Moreover, the overall distribution of CTCAE grades differed significantly between treatment groups, with a greater proportion of severe cisplatin-induced hearing loss among those receiving triweekly cisplatin (χ^2^(3) = 25.76, *P* < 0.0001). Notably, the proportion of patients experiencing grade 3 hearing loss was nearly 3 times higher in the triweekly group (41%) than in the weekly group (14%). This distribution highlights a significant shift toward more severe ototoxic outcomes with the high-dose, triweekly regimen.

**Figure 4:**
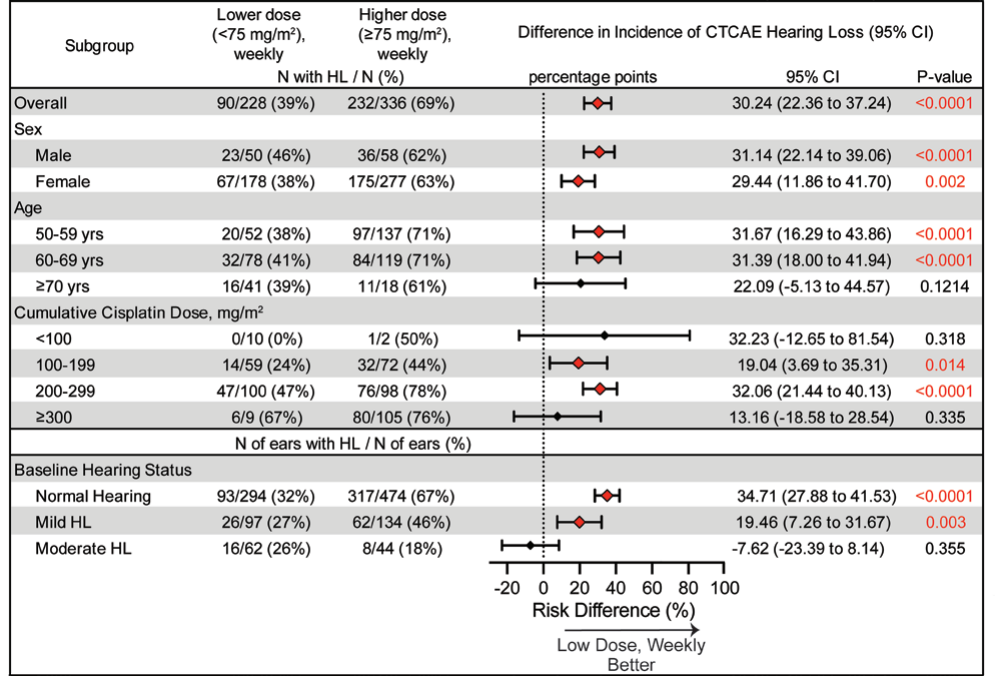
Weekly administration of cisplatin significantly reduces the risk of developing a clinically meaningful cisplatin-induced hearing loss. Analysis of the incidence of a CTCAE grade ≤1 hearing loss for the full cohort and different subgroups. For the overall cohort and the subgroups, the difference in the incidence (%) and 95% CIs were estimated using a stratified risk difference analysis. Significant differences (red diamonds) in the calculated incidence of a hearing loss (HL) were observed for the full cohort, as well as for the male and female subgroups; individuals 50–59 and 60–69 years; individuals receiving total cumulative cisplatin doses 100–199 and 200–299 mg/m^2^; and for those with normal hearing and mild hearing loss at baseline. Red indicates significant *P* values.

The low-dose, weekly cisplatin schedule consistently resulted in reduced hearing loss across subgroups (Figure 4). Overall, 69% (232 of 336) of individuals in the triweekly group developed hearing loss, compared with 39% (90/228) in the weekly group. Statistically significant reductions in hearing loss with weekly treatment were observed among male participants (46% vs. 62%, *P* < 0.001); female participants (38% vs. 63%, *P* < 0.001); individuals aged 50–59 (39% vs. 71%, *P* < 0.001); individuals aged 60–69 (41% vs. 71%, *P* < 0.001); those who received cumulative cisplatin doses of 100–199 mg/m² (24% vs. 44%, *P* < 0.02) and 200–299 mg/m² (47% vs. 78%, *P* < 0.001); and participants with either normal baseline hearing (32% vs. 67%, *P* < 0.001) or mild hearing loss at baseline (27% vs. 46%, *P* < 0.003).

**Figure 5:**
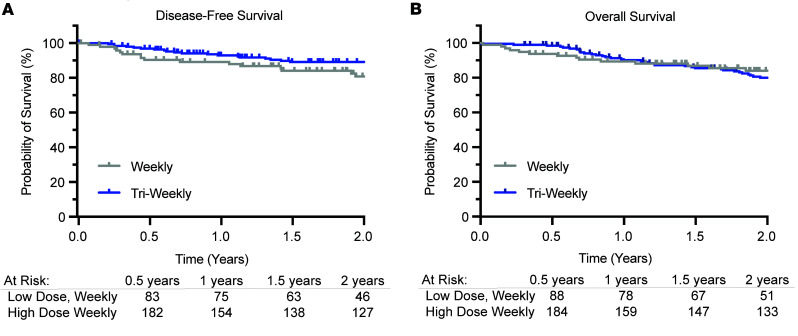
Two-year DSF and OS are not different among individuals receiving weekly or triweekly cisplatin. Kaplan-Meier estimates of disease-free (**A**) and overall (**B**) survival. A log-rank (Mantel-Cox) test indicated no significant difference in either disease-free or OS among groups (*P* > 0.05). Weekly group, *n* = 96; triweekly group, *n* = 192.

Two-year OS and DSF were comparable between the dosing schedules. To determine whether dose and schedule impact treatment efficacy, we examined OS and DFS in available datasets from University of Rochester Medical Center (URMC; *n* = 129) and Washington University in St. Louis (WUSTL; *n* = 160) (Figure 5, A and B). At 2 years, OS exceeded 80% across the cohort (Figure 5A). Median survival could not be calculated due to censoring. However, log-rank (Mantel-Cox) tests revealed no significant differences in OS (χ^2^(1) = 0.214, *P* > 0.05) or DFS (χ^2^(1) = 3.102, *P* > 0.05) between individuals receiving weekly versus triweekly cisplatin.

## Discussion

We found that a low-dose, weekly cisplatin administration schedule was associated with a significantly lower incidence and severity of cisplatin-induced hearing loss compared with a triweekly regimen in patients with HNSCC. Using both ASHA and CTCAE criteria to define clinically meaningful changes in hearing, we observed reductions in incidence from 82% to 57% and from 69% to 39%, respectively, with weekly dosing. Importantly, this reduced toxicity was not detrimental to oncologic efficacy, as 2-year OS and DSF were comparable between the treatment groups.

Cisplatin remains a cornerstone in concurrent CRT for head and neck cancers due to its proven survival benefits in both definitive and adjuvant settings (5, 18, 19). However, its dose-limiting toxicities — including irreversible sensorineural hearing loss — have prompted increasing interest in optimizing dosing schedules to reduce harm without compromising oncologic efficacy (20–22). Li et al. and Gamez et al. indicated that low-dose, weekly administration might result in lower incidences of ototoxicity compared with high-dose, triweekly regimens, though they noted that further validation in larger cohorts was needed (21, 23).

Using both ASHA and CTCAE criteria, we observed a significantly lower incidence of hearing loss in patients receiving low-dose, weekly cisplatin (39%) compared with those receiving triweekly cisplatin (69%) (Figure 3). This finding echoes those of Zuur et al., who reported a markedly lower ototoxicity incidence (31%) with daily cisplatin (6 mg/m²) and accelerated radiation therapy relative to higher-dose regimens (78%) (20). Similarly, Gamez et al. conducted a prospective study comparing weekly (40 mg/m²) and triweekly (100 mg/m²) cisplatin in conjunction with cochlea-sparing intensity-modulated radiation therapy (IMRT) (21). Their results showed that only 13% of patients in the weekly group developed CTCAE grade ≥3 ototoxicity versus 56% in the triweekly group — a trend mirrored in our data, with 14% of patients in the weekly group and 41% in the triweekly group meeting CTCAE grade ≥3 criteria. Together, our findings underscore that not all hearing loss is equivalent in severity. The CTCAE grade distribution revealed that grade 3 hearing loss — indicating the most severe threshold shifts — was substantially more prevalent in the high-dose, triweekly group. This suggests that while low-dose, weekly cisplatin may not eliminate ototoxicity altogether, it may reduce the risk of more debilitating hearing loss, thereby offering a meaningful clinical benefit in terms of preserving auditory function.

Importantly, our study extended follow-up to 2 years and incorporated survival data. We demonstrated that reduced hearing loss with weekly cisplatin did not come at the cost of oncologic control; there were no statistically significant differences in either OS or DSF between the 2 regimens (Figure 5). These results are consistent with phase III trials and meta-analyses that have shown comparable survival with weekly versus triweekly cisplatin regimens (15, 18, 24–26), though many of these prior studies did not focus on audiologic outcomes. Overall high survival rates reported in our dataset are likely due to the large proportion of patients with human papillomavirus–related oropharyngeal cancer, which portends a favorable prognosis and fewer events for comparison.

Systematic reviews (25, 27) and real-world observational studies (26, 28, 29) have suggested that weekly dosing may offer improved tolerability, with fewer treatment interruptions and hospitalizations. Nevertheless, these studies often lacked gold-standard frequency-specific hearing assessments. Our study contributes to filling this gap by analyzing threshold shifts across frequencies and ears and by incorporating both functional (CTCAE) and audiometric (ASHA) criteria for assessment of cisplatin-induced hearing loss. Subgroup analyses further reinforced the robustness of our findings. Weekly cisplatin was associated with reduced hearing loss across age groups, sexes, cumulative dose strata, and baseline hearing status (Figure 4). These trends suggest that the reduced ototoxicity associated with lower, more frequent dosing may be generalizable to a broad population of patients with HNSCC, including those with preexisting vulnerability to hearing loss.

While some centers are still using high-dose cisplatin regimens, many have transitioned to low-dose, weekly administration according to preferences of the treating oncologists and anecdotal evidence of lower overall toxicity. Other centers use both regimens, reserving the high-dose regimen for patients with more advanced head and neck cancers. Our findings support the use of weekly cisplatin where feasible. The balance between toxicity and efficacy becomes especially critical for patients with favorable prognosis, for whom hearing preservation is vital to long-term quality of life. Future studies should consider stratifying patients by ototoxicity risk or using pharmacogenetic screening to tailor cisplatin dosing (23, 30, 31). Moreover, the inclusion of patient-reported hearing outcomes, quality-of-life measures, and real-time audiologic monitoring would provide a more comprehensive view of treatment burden. There is also a need to refine predictive models for cisplatin-induced hearing loss and to explore adjunctive therapies, such as sodium thiosulfate or statins, that could further reduce risk in vulnerable patients (6, 9, 32). Ongoing clinical trials are investigating the protective effects of sodium thiosulfate (NCT05129748, NCT05382338) and statins (NCT04915183) against cisplatin-induced ototoxicity.

Strengths of this study included the large sample size, standardized audiometric evaluations with a defined time point for baseline evaluation (≤120 days prior to start of CRT), use of 2 independent criteria for defining hearing loss, and the inclusion of subgroup analyses and survival data. These features allowed for a more nuanced understanding of the impact of treatment schedule on ototoxicity and patient outcomes. However, several limitations must be acknowledged. This was a primarily retrospective observational study, and although we adjusted for key covariates, unmeasured confounding variables may still be present. While our study did not incorporate the potential contribution of non-cisplatin ototoxic agents, cisplatin-based CRT regimens for head and neck cancer are generally administered as monotherapy with radiation. The absence of detailed concurrent medication records is a limitation; however, standard clinical protocols do not typically include other ototoxic drugs in these treatment regimens. The assignment of patients to dosing schedules was not randomized and may reflect underlying clinical factors. Moreover, individual cisplatin dose was not analyzed as a continuous variable due to intra-patient variability in dosing across cycles within the same treatment schedule. Additionally, we did not include patient-reported outcomes or functional hearing assessments, which could complement the audiometric findings. Survival analyses were limited by censoring and incomplete follow-up in a subset of patients in addition to an overall favorable patient population with a high proportion of oropharyngeal cancer. Finally, while <75 mg/m² versus ≥75 mg/m² is a commonly used clinical threshold, these categories may not fully capture the pharmacologic complexity of cisplatin dosing.

### Conclusion.

Our findings, together with consistent evidence from randomized trials and real-world studies, underscore that weekly cisplatin dosing is not merely a convenient or lower-intensity alternative, but may offer a substantial reduction in the risk of permanent hearing loss without compromising oncologic outcomes. While treatment decisions must ultimately be guided by oncology teams, our data support the consideration of weekly dosing as a viable and potentially otoprotective approach in the management of HNSCC.

## Methods

### Sex as a biological variable.

This retrospective cohort comprised adult patients with head and neck cancer of both biological sexes; however, the sample was predominantly male, consistent with the known higher incidence of this malignancy in men. Sex was treated a priori as a potential confounder and included as a covariate in the multivariable models estimating absolute risk differences and their 95 % confidence intervals.

### Overview.

This study utilized both male and female clinical data from the Enhancing Cancer Hearing Outcomes (ECHO) dataset, developed at the National Institute on Deafness and Other Communication Disorders. The dataset includes combined retrospective and prospective observational data from 5 clinical sites. Retrospective clinical data were obtained from ototoxicity monitoring programs at the University of Rochester Medical Center (URMC, *n* = 233), Walter Reed National Military Medical Center (WRNMMC, *n* = 31), Washington University in St. Louis (WUSTL, *n* = 201), and the Stanford Ear Institute (SEI, *n* = 508). Prospective data were collected through an observational study conducted at the NIH in partnership with the Johns Hopkins University (JHU) Departments of Otolaryngology–Head and Neck Surgery and Radiation Oncology and Molecular Sciences (JH/NIH, *n* = 28). All datasets were harmonized to ensure consistency in variable definitions and facilitate integrated analysis. Data were filtered to include adults (≥18 years) diagnosed with HNSCC treated with cisplatin-based CRT. Inclusion criteria included availability of baseline audiograms obtained within 120 days prior to cisplatin initiation and posttreatment audiograms obtained within 120 days after all cycles of cisplatin were completed. Participant demographics (age, sex, baseline hearing status) and clinical characteristics (cancer site, stage, treatment schedule) are summarized in Table 1.

### Data analyses.

The primary outcome was the incidence of hearing loss as defined by 2 established ototoxicity grading systems: ASHA (1994) and CTCAE v5.0 (NCI, 2017). Threshold shifts were calculated as the difference in decibels hearing level (dB HL) between pre- and posttreatment audiograms at each frequency. In instances where there was no measurable response at the highest presentation level of the audiometer, 5 dB were added to that level in order to estimate the threshold for the purpose of calculating threshold shifts. ASHA criteria define a clinically significant change as either a ≥10 dB shift at 2 consecutive frequencies or a ≥20 dB shift at a single frequency in at least one ear (ASHA, 1994). CTCAE v5.0 defines severity using the following grades for 1–8 kHz frequencies (NCI, 2017): grade 1 (mild), 15–25 dB averaged threshold shift at 2 contiguous frequencies; grade 2 (moderate), >25 dB averaged shift at 2 contiguous frequencies; grade 3 (severe), >25 dB averaged shift at 3 contiguous frequencies

CTCAE scores were assigned per individual based on meeting the specified criteria in at least one ear. To further characterize ototoxicity, stratified analyses were conducted across CTCAE grades 1, 2, and 3 to assess differences in severity between cisplatin dosing schedules. This approach evaluated whether hearing loss occurred, but also the extent of impairment, in the event that weekly cisplatin reduced severity without fully preventing toxicity

The secondary outcome was the change in hearing thresholds per ear across standard audiometric frequencies (0.25–4 kHz), used to assess the influence of covariates such as cisplatin schedule, age, sex, cumulative cisplatin dose, and baseline hearing status. Hearing status at baseline was based on the PTA of 1, 2, and 4 kHz in dB HL: normal (PTA ≤20), mild (PTA >20, <40), moderate (PTA ≥40, ≤70), severe (PTA >70, <90), profound (PTA≥90) (17).

### Statistics.

Two-way ANOVA with Šidák’s post hoc correction (GraphPad Prism 10) was used to compare hearing threshold shifts by frequency across treatment groups (weekly vs. triweekly cisplatin). The incidence of ASHA- and CTCAE-defined hearing loss was treated as categorical data and analyzed using χ^2^ tests (PROC FREQ v9.4, SAS).

To estimate the absolute risk difference in CTCAE-defined hearing loss (grade ≥1) between treatment groups, we used a stratified risk difference analysis. Stratification was based on cisplatin schedule. Risk differences and 95% CIs were calculated in SAS using the RISKDIFF option in PROC FREQ. Subgroup analyses were performed for age, sex, cumulative dose categories, and baseline hearing status (normal, mild, moderate). For each subgroup, 2 × 2 contingency tables were created, and χ^2^ tests of independence assessed the significance of associations between treatment schedule and hearing loss.

For our secondary outcome, we performed OLS regression using PROC REG. The dependent variable was the posttreatment PTA shift from 0.5 to 4 kHz. Independent variables included cisplatin schedule (categorical), age (continuous), sex (categorical), cumulative cisplatin dose (continuous), and baseline hearing PTA 0.5-4 kHz (continuous). Both univariate and multivariate models were fit; 95% CIs for parameter estimates were calculated using the CLB option. Model performance was assessed using *r^2^* and adjusted *r^2^*statistics.

Survival analyses were performed using Kaplan-Meier estimates for OS and DSF over 2 years after treatment. Data were available from URMC (*n* = 129) and WUSTL (*n* = 160). Survival curves were generated in GraphPad Prism 10, and comparisons between cisplatin schedules were made using the log-rank (Mantel-Cox) test.

All statistical analyses were conducted using SAS v9.4 (SAS Institute Inc.) and GraphPad Prism 10. A *P* value less than 0.05 was considered statistically significant.

### Study approval.

Study procedures were approved by the IRBs of the participating sites: University of Rochester (RSRB00060424), Washington University in St. Louis (IRB#20220516), Stanford University (IRB-55582), and the Defense Health Agency Human Research Protections Program (DSA 876612). Prospective components were approved by the NIH IRB (IRB 17-DC-0138) and were registered at ClinicalTrials.gov (NCT03225157). Written informed consent was obtained from all participants enrolled in the prospective component.

### Data availability.

Values for all data points in graphs are reported in the Supporting Data Values file. Data will be made available upon request. The Enhancing Cancer Hearing Outcomes (ECHO) dataset requires a data share agreement and is available upon request from the corresponding author.

## Author contributions

KAF and LLC designed the study. Data collection was conducted by PDA, MHC, KAF, CM, CEO, DSL, PLSM, and PK. BRP, SDN, NCS, DAM, PSM, PK, and SAK provided patient management and referral and/or patient care. KAF, AC, and AB conducted statistical analysis, and figures were generated by KAF, ASC, and AB. KAF, AB, ASC, and LLC interpreted the data. The manuscript was prepared by KAF, ASC, AB, and LLC and reviewed by all coauthors. KAF and LLC are guarantors or this work, and as such had full access to all of the data in the study and take responsibility for the integrity of the data and accuracy of the data analysis.

## Funding support

This work is the result of NIH funding, in whole or in part, and is subject to the NIH Public Access Policy. Through acceptance of this federal funding, the NIH has been given a right to make the work publicly available in PubMed Central.

Division of Intramural Research at the National Institute on Deafness and Other Communication Disorders (NIDCD), NIH, project 1ZIADC000079.

## Supplementary Material

ICMJE disclosure forms

Supporting data values

## Figures and Tables

**Table 1:**
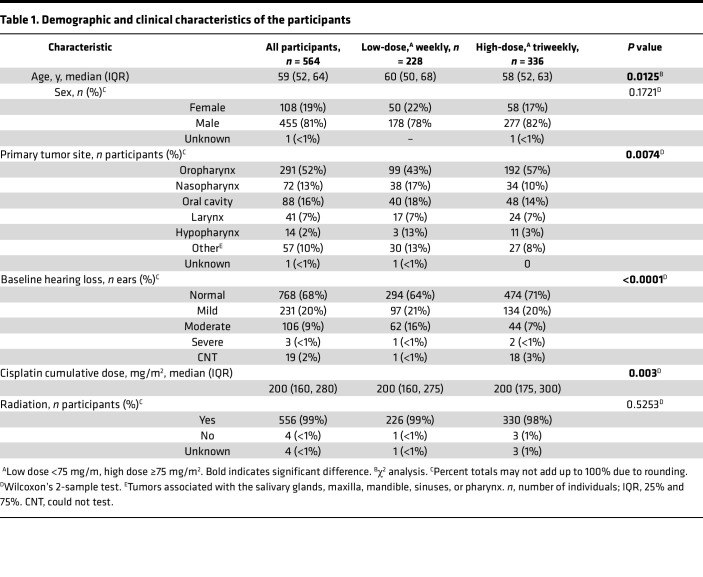
Demographic and clinical characteristics of the participants

**Table 2:**
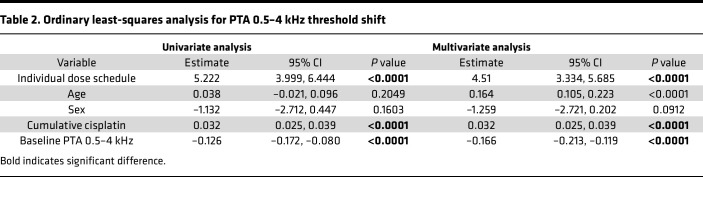
Ordinary least-squares analysis for PTA 0.5–4 kHz threshold shift

## Version 1 10/22/2025

Electronic publication
